# Functions and Interactions of Mammalian KDM5 Demethylases

**DOI:** 10.3389/fgene.2022.906662

**Published:** 2022-07-11

**Authors:** Egor Pavlenko, Till Ruengeler, Paulina Engel, Simon Poepsel

**Affiliations:** ^1^ University of Cologne, Center for Molecular Medicine Cologne (CMMC), Faculty of Medicine and University Hospital, Cologne, Germany; ^2^ Cologne Excellence Cluster for Cellular Stress Responses in Ageing-Associated Diseases (CECAD), University of Cologne, Cologne, Germany

**Keywords:** KDM5, gene regulation, epigenetics, histone demethylation, JmjC oxygenases

## Abstract

Mammalian histone demethylases of the KDM5 family are mediators of gene expression dynamics during developmental, cellular differentiation, and other nuclear processes. They belong to the large group of JmjC domain containing, 2-oxoglutarate (2-OG) dependent oxygenases and target methylated lysine 4 of histone H3 (H3K4me1/2/3), an epigenetic mark associated with active transcription. In recent years, KDM5 demethylases have gained increasing attention due to their misregulation in many cancer entities and are intensively explored as therapeutic targets. Despite these implications, the molecular basis of KDM5 function has so far remained only poorly understood. Little is known about mechanisms of nucleosome recognition, the recruitment to genomic targets, as well as the local regulation of demethylase activity. Experimental evidence suggests close physical and functional interactions with epigenetic regulators such as histone deacetylase (HDAC) containing complexes, as well as the retinoblastoma protein (RB). To understand the regulation of KDM5 proteins in the context of chromatin, these interactions have to be taken into account. Here, we review the current state of knowledge on KDM5 function, with a particular emphasis on molecular interactions and their potential implications. We will discuss and outline open questions that need to be addressed to better understand histone demethylation and potential demethylation-independent functions of KDM5s. Addressing these questions will increase our understanding of histone demethylation and allow us to develop strategies to target individual KDM5 enzymes in specific biological and disease contexts.

## Introduction

Chromatin structure and its chemical modifications are central to the coordination of transcriptional activity and other nuclear processes. Post-translational modifications (PTMs) of histone proteins that form the core of nucleosomes, the basic organizing unit of chromatin, are key in these processes and tightly linked to chromatin regulation (Strahl and Allis, 2000). Histone PTMs are markers of regulatory genomic elements and functional chromatin states. Accordingly, the prevalence of histone PTMs is highly dynamic and reflects cellular states and their transitions. For example, during cellular differentiation, the landscape of histone PTMs undergoes characteristic changes that correlate with the re-shaping of transcription patterns (Li et al., 2007). A key notion in epigenetics is that histone PTMs are introduced and removed by enzymes that act in a spatio-temporally defined manner. Thus, their faithful regulation is required for normal development and cellular differentiation (Margueron and Reinberg, 2011). Protein domains that specifically recognize histone PTMs, so-called ‘reader’ domains, are important for these regulatory mechanisms. Reader domains recruit associated proteins and multi-protein complexes to their genomic targets, but also couple recruitment to local allosteric activation or inhibition of associated enzymes (Torres and Fujimori, 2015). The assembly, composition, and dynamic chromatin interactions of multi-subunit complexes give rise to the complexity of chromatin regulation that is still only beginning to be elucidated. Key to these intricate mechanisms are the interactions to recruit and locally regulate chromatin modifying enzymes as well as their dynamic interplay to control chromatin structure, transcription and other processes.

Deciphering the diverse roles of histone PTMs in different biological contexts remains a substantial challenge and thus is subject of intense research. While detailed molecular mechanisms and implications remain poorly understood in many instances, the most prevalent histone PTMs are reasonably well described. Methylation of lysine 4 of histone H3 (H3K4me1/2/3) is generally associated with genomic regions marked by high transcriptional activity. Alternatively, when present alongside trimethylated lysine 27 of histone H3 (H3K27me3), this PTM is associated with a poised state allowing for rapid transcriptional activation or repression, particularly during early development (Santos-Rosa et al., 2002; Heintzman et al., 2007; Kim and Buratowski, 2009; Rada-Iglesias et al., 2011). Accordingly, factors that interact with methylated H3K4 are involved in transcriptional regulation, such as general transcription factors (Vermeulen et al., 2007), chromatin remodelers such as the BAF and NURF complexes (Wysocka et al., 2006; Local et al., 2018) or methyltransferase complexes such as KMT2 (Park et al., 2010; Eberl et al., 2013).

## Activity and Functions of KDM5 Demethylases

**FIGURE 1:**
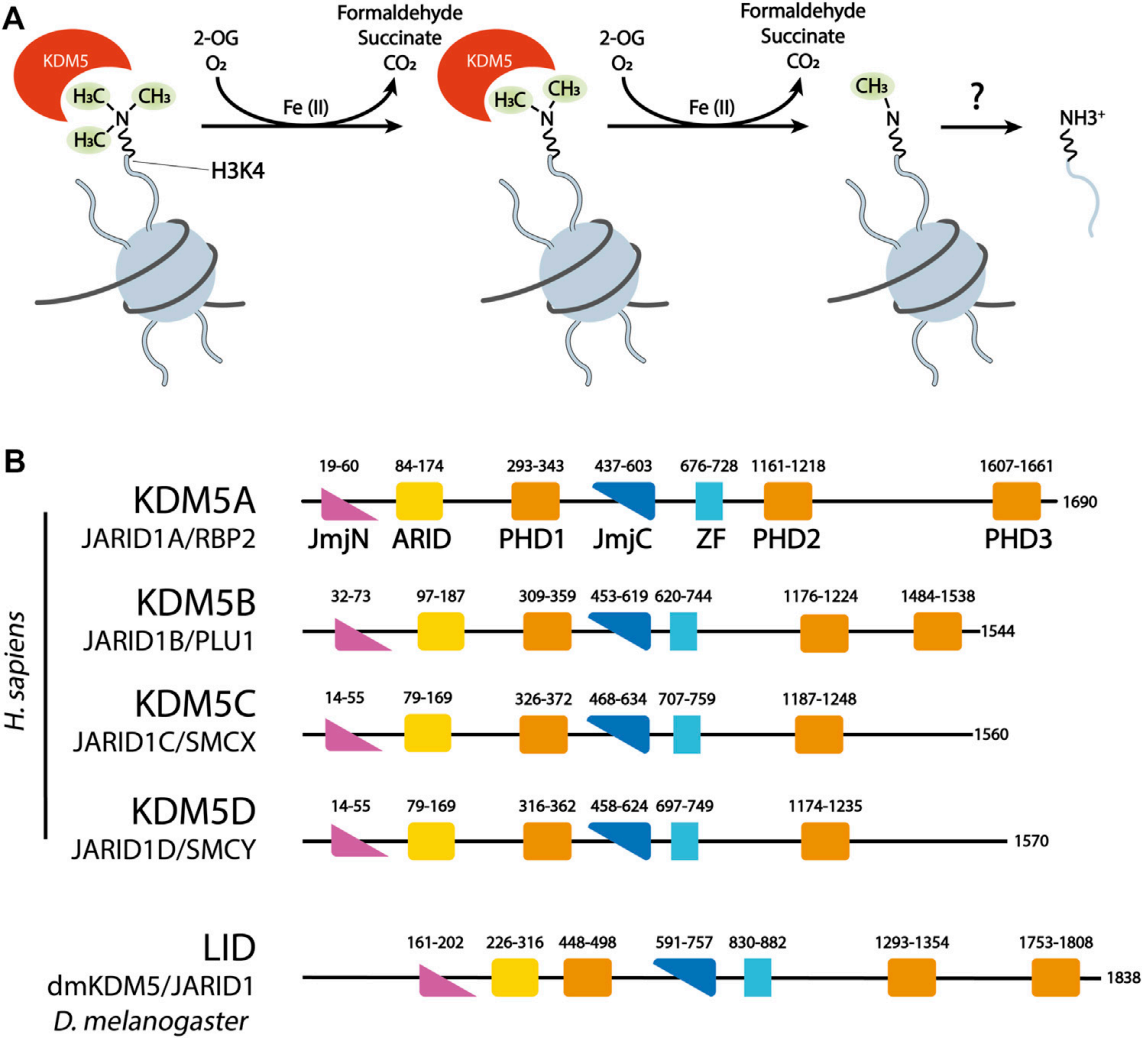
**(A)** KDM5 demethylases remove methyl groups from H3K4 in a sequential manner, using the dioxygenase activity of their catalytic JmjC domain. 2-Oxoglutarate (2-OG) is decarboxylated to succinate. Formaldehyde, one of the products of demethylation, is commonly detected in quantitative assays of JmjC demethylase activity. **(B)** Domain organization of the four human KDM5 demethylases and the *Drosophila* KDM5 homolog Lid. ZF = C5HC2 Zinc Finger. Numbers correspond to the amino acid numbering of each KDM5 protein.

The four human members of the KDM5 family, KDM5A-D, each of which has a highly similar mouse homolog, are part of a large group of Jumonji C (JmjC) domain containing, 2-oxoglutarate (2-OG)- and Fe(II)-dependent dioxygenases that comprises numerous enzymes, among them many with chromatin associated functions. Interestingly, the biological function of JmjC domain dioxygenases, as well as their use of and responsiveness to metabolites such as 2-OG, fumarate and succinate, mediate key roles in cancer biology, in particular cancer metabolism (Xu et al., 2011; Losman et al., 2020). The idea that JmjC dioxygenases may have histone lysine demethylating activities was based on the discoveries of DNA demethylation by the dioxygenase AlkB (Trewick et al., 2002), and the hydroxylation of hypoxia-inducible factor (HIF) by EGLN (Bruick and McKnight, 2001; Jaakkola et al., 2001). Indeed, following the first report of a JmjC domain histone demethylase (Tsukada et al., 2006), all four human KDM5 enzymes were shown to specifically demethylate lysine 4 of histone H3 (H3K4) in a series of landmark studies (Christensen et al., 2007; Iwase et al., 2007; Klose et al., 2007; Seward et al., 2007; Tahiliani et al., 2007). The catalytic activity of JmjC domain demethylases involves the decarboxylation of the cofactor 2-OG to succinate and CO_2_, as well as the hydroxylation of methylated lysine, leading to the spontaneous decomposition of an unstable hemi-aminal intermediate into demethylated lysine and formaldehyde (Walport et al., 2012) (Figure 1A). KDM5 demethylases are generally considered to specifically demethylate the di- and trimethylated state of H3K4 (H3K4me2/3), leading to the hypothesis that the coordination with the activity of the H3K4me1/2-specific demethylase LSD1 may be required for the complete demethylation of H3K4 (Christensen et al., 2007; Klose et al., 2007; Seward et al., 2007; Tahiliani et al., 2007). However, *in vitro* data suggests that demethylation of H3K4me1 by KDM5 enzymes is also possible (Metzger et al., 2010; Kristensen et al., 2012). How specific targeting of different methylation states of H3K4 is brought about, and whether there are mechanisms regulating this specificity is currently unknown.

Members of the KDM5 family of proteins had been known to perform regulatory roles in transcription before their demethylase activity was established. For example, an early report described KDM5B as a co-repressor of developmental transcription factors such as paired box 9 (PAX9) and brain-factor 1 (BF-1) (Tan et al., 2003). Since H3K4 methylation had been recognized as a feature of active chromatin (Litt et al., 2001), an obvious mechanism of KDM5 enzymes was the demethylation of H3K4me2/3 facilitating transcriptional repression. In agreement with this hypothesis, human KDM5 proteins were shown to cause an overall decrease in cellular levels of H3K4me3 when overexpressed (Christensen et al., 2007; Iwase et al., 2007; Klose et al., 2007). Aspects of KDM5 function, such as HOX gene repression by KDM5A (Christensen et al., 2007) and promotion of neuronal viability by KDM5C (Iwase et al., 2007), could be directly linked to their demethylase activity. However, it was also noted in these early studies that KDM5 function may partly be mediated independently of their catalytic activity. For example, KDM5A knock-out mouse embryonic fibroblasts did display transcriptional repression of KDM5A targets even when a catalytically inactive KDM5A was expressed (Klose et al., 2007).

A growing body of literature illustrates the diverse roles of KDM5 demethylases in gene regulation, differentiation and developmental processes. KDM5 proteins help to control cellular differentiation in a number of contexts, but the reported mechanisms and implications vary and seem contradictory at times. For example, loss of KDM5B is associated with embryonic stem cell (ESC) differentiation *in vitro* (Xie et al., 2011) and was shown to antagonize terminal ESC differentiation by balancing cell proliferation and differentiation (Dey et al., 2008). At the same time, the enzyme was required for neuronal differentiation in another study (Schmitz et al., 2011). All three studies have in common, however, that lineage-specific gene expression during differentiation was impaired upon KDM5 depletion (Dey et al., 2008; Schmitz et al., 2011; Xie et al., 2011). In the context of its interactions with the retinoblastoma protein (RB) it was suggested that KDM5A can contribute to the transcriptional activation of genes involved in cellular differentiation (Benevolenskaya et al., 2005), illustrating that KDM5 function is not limited to repressive effects on transcription. KDM5 enzymes have also been implicated in cell cycle control. For example, KDM5A and C genomic occupancy and demethylase activity were required for transcriptional activity of cell cycle regulators during adipocyte differentiation (Brier et al., 2017). The observation that, in the same experimental system, other genes marked by low H3K4me3 levels at their promoters were repressed by KDM5s, underscores the significance of cellular and genomic context for the implications of KDM5 occupancy and activity.

The single KDM5 homologs in *Drosophila melanogaster* and *Caenorhabditis elegans*, called Little imaginal discs (Lid) and retinoblastoma binding protein related 2 (RBR-2), respectively, are required for normal development (Gildea et al., 2000; Christensen et al., 2007). Mammalian KDM5 enzymes show distinct developmental defects upon their deletion, hinting at specific and partially non-redundant roles of these proteins in development. For example, loss of KDM5B leads to defects of respiratory function and neuronal development in mice (Albert et al., 2013). Furthermore, KDM5 enzymes were shown to be involved in DNA replication (Liang et al., 2011; Rondinelli et al., 2015; Gaillard et al., 2021), DNA repair (Gong et al., 2017; Kumbhar et al., 2021) and metabolic pathways (Chang et al., 2019). Comprehensive reviews discuss the functions of KDM5 and other demethylases in development and differentiation in more detail (Pedersen and Helin, 2010; Kooistra and Helin, 2012; Dimitrova et al., 2015; Punnia-Moorthy et al., 2021).

## KDM5 Demethylases in Human Diseases

A number of observations provide evidence of a critical role of KDM5 demethylases in diverse disease settings. For instance, KDM5C mutations are frequently found in X-linked intellectual disability (Jensen et al., 2005; Hatch and Secombe, 2021), linking KDM5C function to developmental regulation. Aberrant levels, in particular the amplification and/or overexpression of KDM5 demethylases in many types of cancer, including gastric (Zeng et al., 2010), breast (Yamane et al., 2007; Yamamoto et al., 2014), prostate (Xiang et al.,2007), lung cancer (Oser et al., 2019) and leukemia (Xue et al., 2020) strongly link KDM5 demethylases to cancer biology. KDM5C was identified as a potential cancer driver (Bailey et al., 2018), and KDM5 inhibition has a strong inhibitory effect on tumor growth in tissue culture and *in vivo* models (Yamane et al., 2007; Vinogradova et al., 2016; Vogel et al., 2019). In some instances, specific roles have been identified by which KDM5 demethylases control tumor phenotypes and therapeutic response. Both KDM5A and KDM5B have been shown to be key determinants of a dynamic, phenotypic heterogeneity in cancer, defining differentiation, proliferation and responsiveness of cell populations to therapeutic intervention. One observation was a marked transcriptional heterogeneity of cancer cells depending on KDM5A and B functions (Hinohara et al., 2018). KDM5A was further identified as a critical factor characterizing drug tolerant persister cancer cells that mediated intrinsic resistance towards chemotherapy in a non-small cell lung cancer (SCLC) cell line (Sharma et al., 2010; Vinogradova et al., 2016). Melanoma cells were shown to be composed of heterogeneous cancer cells that, when expressing high levels of KDM5B, are resistant to therapy such as MAPK inhibition, giving rise to tumor repopulation after initial therapy (Roesch et al., 2010). KDM5B was also identified as a regulator of cancer stem cell properties in oral cancers (Facompre et al., 2016). These studies established KDM5 demethylases as regulators of epigenetic plasticity in human cells that are likely to be of significant interest for future drug development efforts.

In addition, several other mechanisms have been suggested to underlie KDM5 involvement in cancer. By participating in DNA damage response pathways, some KDM5 demethylases may be important mediators of genome stability, for example in renal cancer (Li et al., 2014; Gong et al., 2017). In melanoma, KDM5B was shown to induce an anti-tumor immune response and was required for immune evasion of cells in an *in vivo* model (Zhang et al., 2021). Moreover, KDM5 demethylases are involved in cell cycle regulation (Hou et al., 2012), invasion (Teng et al., 2013), differentiation (Oser et al., 2019) and metabolism (Roesch et al., 2013) of cancer cells. Taken together, KDM5 demethylases perform diverse roles that in many cases favor the pathogenesis and therapy resistance of various cancers. At the same time, the observed complexity of KDM5 functions strongly suggests that KDM5 activities may also serve tumor suppressive functions in some instances (Li et al., 2016a), e.g., facilitating genome stability (Li et al., 2014), underlining the need to understand the underlying mechanisms for context-dependent KDM5 targeting by therapeutic agents. The accumulating evidence of KDM5 function in cancer is discussed in detail in a number of excellent, recent reviews (Hojfeldt et al., 2013; Johansson et al., 2016; Harmeyer et al., 2017; Plch et al., 2019; Yang et al., 2021). As a consequence of the above findings, there has been an increasing interest in developing potent and specific inhibitors against KDM5 demethylases for use in a clinical setting (Johansson et al., 2016; Kaniskan et al., 2018). Major obstacles remain to be addressed on the way towards efficient and specific therapeutic approaches targeting KDM5s. For example, KDM5 inhibitors are mostly competitors of the cofactor 2-OG that as a metabolite is present at high concentrations, hampering competitive inhibition (Kaniskan et al., 2018). Moreover, the catalytic domains and 2-OG binding pockets are structurally highly similar within the KDM5 family, leading to difficulties in specifically targeting individual KDM5 enzymes (Horton et al., 2016; Johansson et al., 2016; Vinogradova et al., 2016). Of note, compound screens and activity assays so far have relied on peptide substrates and truncated KDM5 proteins that can be readily purified in amounts required for these high-throughput approaches. However, the binding of their natural chromatin substrates, as well as allosteric regulatory mechanisms may uncover novel targets of small molecules.

## Mechanisms of KDM5 Function

### KDM5 Structure, Chromatin Interactions and Activity Regulation

**FIGURE 2:**
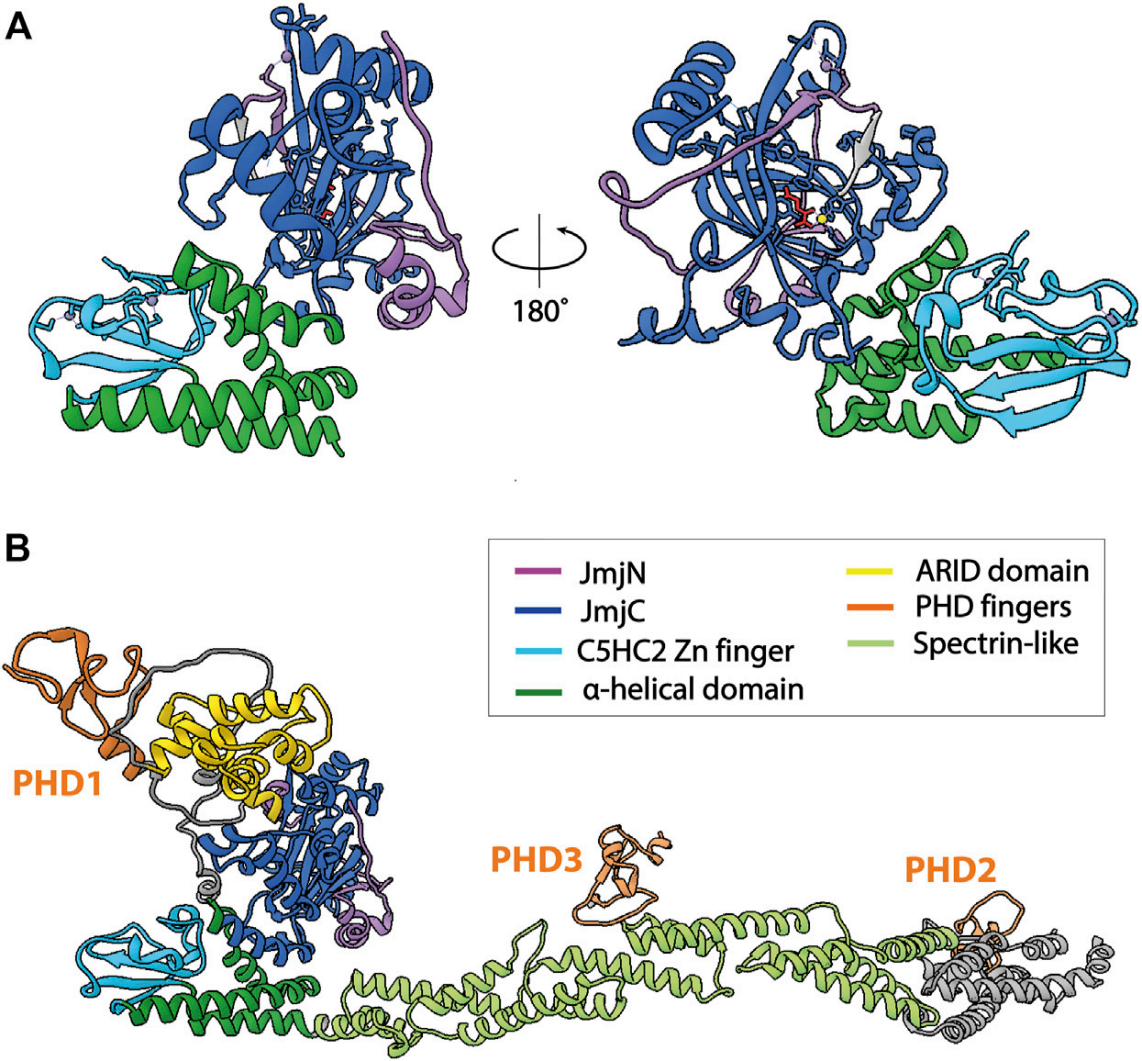
Structures of KDM5B. The catalytic cores of KDM5 enzymes are structurally highly similar, therefore only structures of KDM5B are shown. **(A)** Atomic model of the catalytic core of KDM5B [PDB 5A1F (Johansson et al., 2016)]. The construct crystallized was composed of the JmjN and JmjC domains, as well as the α-helical domain including the C5HC2 Zn finger. The ARID and PHD1 domains were not included. The α-helical domain and the C5HC2 Zn-finger are required for demethylase activity, whereas the ARID and PHD1 domains are dispensable for the demethylation of peptide substrates by truncated KDM5s. **(B)** Alphafold2 prediction of full-length KDM5B (AF-Q9UGL1-F1), showing the predicted arrangement of the protein domains C-terminal of the catalytic core in an extended conformation, in agreement with experimental data (Dorosz et al., 2019). Of note, other conformations cannot be excluded due to the flexibility of the coiled-coil domain. Structural predictions of other KDM5s show a more compact orientation of the C-terminal part, with the PHD2 domain being located in close proximity to the N-terminal, catalytic core. Unstructured regions with low prediction confidence were omitted from the figure for clarity.

KDM5 demethylases are multi-domain proteins that share a common domain architecture. The four human KDM5 family members have an almost identical arrangement of protein domains, with the exception that KDM5C and D lack the most C-terminal plant homeodomain (PHD)—type zinc finger (Figure 1B). Catalytic activity is mediated by a composite JmjN/JmjC domain that, together with a helical domain surrounding a C5HC2 zinc finger motif required for demethylation (Yamane et al., 2007), make up a compact catalytic core (Figure 2A) (Johansson et al., 2016). The DNA binding AT-rich interactive (ARID) and the first PHD domain are partially dispensable for the catalytic activity of a truncated construct of KDM5B in the context of peptidic substrates (Johansson et al., 2016), but likely play important roles in the allosteric regulation of KDM5 demethylase activity (see below and (Klein et al., 2014; Torres et al., 2015)). The catalytic cores of KDM5A, B and C have been explored in detail structurally via x-ray crystallography and functionally with biochemical approaches (Horton et al., 2016; Johansson et al., 2016; Vinogradova et al., 2016). These structures have provided valuable information on the architecture of the active site and surrounding protein domains, and have enabled the detailed analysis of inhibitor binding and their modes of action. Additional structural information is still required on how the substrate histone tail is engaged with the active site, potentially providing an explanation for the requirement of the C5HC2 Zn finger for catalytic activity. The regions C-terminal of the catalytic core are less well described, comprising two to three more PHD domains, as well as a region that is predicted to be rich in α-helices adopting a coiled-coil arrangement (Figure 2B). A structural study of human full-length KDM5B using small-angle X-ray scattering (SAXS), hydrogen deuterium exchange mass spectrometry and negative-stain electron microscopy combined with homology modeling approaches showed that the C-terminal half of the protein indeed displayed a coiled-coil structure (Dorosz et al., 2019). KDM5B was shown to adopt an overall elongated conformation with the catalytic and most C-terminal regions linked flexibly by a coiled-coil, spectrin-like domain. This overall structural arrangement is in agreement with structure predictions using the Alphafold algorithm (Jumper et al., 2021) (Figure 2B).

The PHD1 domain that is positioned C-terminal of the catalytic JmjN/C domains plays an important role in substrate engagement and activity regulation of KDM5A and B. This domain has a binding preference towards unmodified H3 peptides (Zhang et al., 2014) and may also interact with methylated H3K9 (Klein et al., 2014). Interestingly, engagement of H3 peptides unmethylated at K4 confers allosteric activation of KDM5A and B demethylase activities (Klein et al., 2014; Torres et al., 2015). For KDM5A, it was shown that this activation mechanism involves a conformational rearrangement of the active site (Longbotham et al., 2019). The mechanistic details of how this regulation is brought about structurally, in particular in the context of full-length KDM5 enzymes and chromatin substrates, remain to be elucidated. Functionally, since fully demethylated H3K4 is the final product of KDM5 activity, potentially in coordination with the H3K4me1 specific lysine demethylase LSD1, sequestering the product of catalysis may prevent re-methylation of H3K4. The observed allosteric activation could also imply a feed-forward mechanism propagating demethylated H3K4. Similar mechanisms are known for other chromatin modifiers such as Polycomb repressive complex 2 (PRC2) (Margueron et al., 2009; Poepsel et al., 2018). Indeed, H3 tail binding by PHD1 was required for the stimulation of breast cancer cell migration upon KDM5B overexpression (Klein et al., 2014), indicating a physiological relevance of this interaction. The yeast ortholog of KDM5 demethylases, Jhd1, was shown to depend on its PHD domain for chromatin engagement in cells (Huang et al., 2010).

**FIGURE 3:**
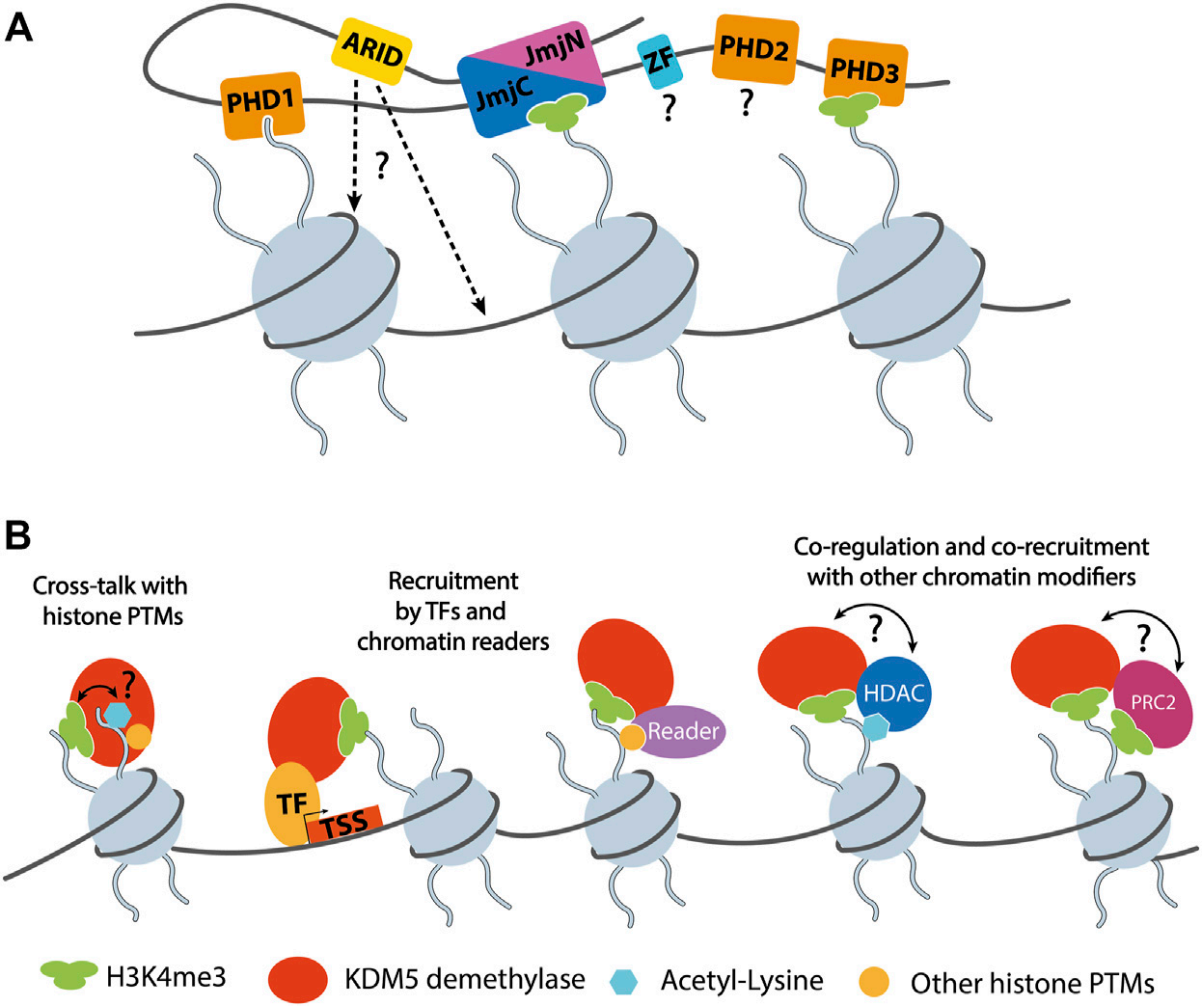
**(A)** Schematic representation of KDM5 interactions with chromatin. The catalytic composite JmjN/C domain binds the substrate H3 tail harboring methylated H3K4 (depicted as H3K4me3 for simplicity). Two of the PHD domains, PHD1 and 3, were shown to interact with unmethylated and trimethylated H3K4, respectively. The ARID domains are known DNA binding domains with a role in KDM5 chromatin targeting. How DNA binding is mediated in the context of full-length KDM5 proteins is currently unknown, since the arrangement of JmjC and ARID domains seems to be incompatible with the binding of nucleosomal DNA, according to homology models (Horton et al., 2016). The roles and potential chromatin interactions of the C5HC2 and PHD2 Zn fingers are unknown. Note that the depiction of three nucleosomes was chosen for clarity. It is not known how many nucleosomes are bound by a single KDM5 protein simultaneously. **(B)** Functional KDM5 interactions on chromatin. So far, only the binding of unmethylated H3K4 has been shown to regulate the demethylase activity of KDM5s. Given the potential interactions with HDACs, a direct or indirect responsiveness to other histone PTMs such as acetylated lysines, is conceivable. KDM5 proteins are recruited by transcription factors (TFs), reader domain proteins, or mediated by the association with other epigenetic regulators such as HDAC complexes or PRC2. The interaction and functional interplay of KDM5s with HDAC complexes and PRC2 suggests a potential mutual regulation of demethylase and other chromatin modifying activities. Such a direct interplay remains to be demonstrated experimentally.

Apart from the active site and PHD1 domains, the PHD3 and ARID domains are likely to contribute to chromatin engagement of KDM5 enzymes (Figure 3A). PHD2 has not yet been biochemically or structurally characterized in detail and did not show histone tail binding. The C-terminal PHD domain of KDM5B was shown to preferentially bind H3K4me2/3, the substrates of KDM5 enzymes, and may therefore play a role in substrate recognition (Klein et al., 2014) (Figure 3A). DNA binding of the ARID domains may serve as an additional anchor point on chromatin. Since the ARID domain is located in the vicinity of the JmjN/C domain, it could be involved in substrate nucleosome recognition (Figure 3A). However, in the conformation that was resolved by X-ray crystallography, DNA binding would be precluded sterically (Horton et al., 2016; Vinogradova et al., 2016), suggesting that, in the context of nucleosomes, the protein may adopt a different conformation compatible with DNA binding. The binding preferences of the KDM5A and B ARID domains have been determined experimentally (Scibetta et al., 2007; Tu et al., 2008) and were shown to be important for H3K4 demethylation by KDM5A in cells (Tu et al., 2008).

Despite detailed structural analyses of individual domains of KDM5 demethylases, their contribution to the activity and function of the complete proteins remain incompletely understood. This is in part due to a lack of high-resolution structural information on full-length KDM5 enzymes. Interactions with chromatin and other binding partners have not been elucidated yet, hampering the investigation of KDM5 function in the context of chromatin. Therefore, it will be key to consider full-length KDM5 for future structural and functional analyses.

KDM5 demethylases take part in nuclear processes as diverse as transcriptional repression, replication and DNA repair (see above). Accordingly, they perform roles within diverse chromatin settings regarding the identity, regulatory state, and function of the respective genomic region. Additionally, KDM5 demethylases, like other chromatin modifying enzymes, function via their catalytic activity or independently of catalysis. These aspects underline the complexity of KDM5 biology, the molecular basis of which has so far remained poorly defined. For example, H3K4me2/3 demethylation can have various consequences depending on the local context. Since H3K4me2/3 is highly enriched in actively transcribed promoter regions, an obvious consequence of demethylation would be reduced transcriptional activity, as was shown in a number of instances (Christensen et al., 2007; Dey et al., 2008). However, H3K4me2/3 removal may also have a positive effect on transcription, e.g. by reducing spurious transcription from within gene bodies, as described for KDM5B, thereby facilitating productive transcriptional elongation and increasing the transcriptional output (Xie et al., 2011). Moreover, changes in H3K4me3 levels not always correlate with transcriptional activation, and association of KDM5B with H3K4me3-bearing promoters was shown to lead to repression or activation, depending on the genomic context (Kidder et al., 2014; Brier et al., 2017). Demethylase activity may also be required for establishing and maintaining PTM configurations specific for functional elements within the genome. For example, a possible product of KDM5 activity, H3K4me1, is a characteristic feature of enhancer regions (Heintzman et al., 2007). While the importance of H3K4me1 for enhancer function is controversial, H3K4 demethylation may help shape its genomic distribution, safeguarding the integrity of epigenetic regulatory pathways. Accordingly, demethylation by KDM5C was shown to facilitate enhancer activity and function through its localized activity at enhancers, potentially through the removal of H3K4me3, thereby reducing aberrant enhancer over-activation (Outchkourov et al., 2013; Shen et al., 2016). A similar role in maintaining the functional integrity of gene regulatory elements was shown for KDM5B controlling the local distribution of H3K4me3 in mouse ESCs. Consequently, loss of KDM5B in this system compromised promoter and enhancer function, as well as transcriptional dynamics during differentiation (Kidder et al., 2014).

In summary, mechanistic knowledge on KDM5 demethylase activity, regulation and function is still relatively scarce, despite their implications in key cellular processes and disease. Defining mechanisms of recruitment, chromatin engagement and activity will be essential to decipher how the diverse roles of KDM5 demethylases are controlled. In addition to their internal structure and interactions with nucleosomal substrates and DNA, intermolecular interactions with other chromatin associated factors are of key importance for KDM5 function. Our current knowledge of how these interactions impact KDM5 function will therefore be reviewed below.

## Interactions of KDM5 Demethylases

### Retinoblastoma Protein

The retinoblastoma protein (RB) was the first known interaction partner of KDM5 demethylases. In fact, KDM5A was initially identified in a screen for RB binders, hence the name RB binding protein 2 (RBP2) (Defeojones et al., 1991). Since then, a number of studies have explored their functional relationship. RB is best known as a tumor suppressor dysfunctional in many types of human cancers including retinoblastoma (Friend et al., 1986), breast (Lee et al., 1988), and lung cancer (Harbour et al., 1988). Consequently, intense research has been addressing its function, particularly in cell cycle regulation. RB prevents progression from G1 to S phase (Weinberg, 1995) by binding and inhibiting E2F transcription factors (TFs), leading to the repression of E2F target genes and ultimately inducing cell cycle arrest. RB interactions depend on the phosphorylation state of its multiple phosphorylation sites. Hypophosphorylated RB is associated with an active state competent of blocking cell cycle progression. Upon phosphorylation by cyclin-dependent kinases (CDKs), RB releases E2F inhibition, allowing for cell cycle progression (Chen et al., 1989; Harbour et al., 1999). Besides the hyperphosphorylated, inactive state, individual phosphorylation events can modulate RB structure, interactions, and specific functions (Sanidas et al., 2019).

While early research largely focused on its impact on cell proliferation, it has since become clear that RB is involved in a multitude of other processes through E2F-dependent or -independent mechanisms. For example, RB is involved in DNA repair, replication, apoptosis, and the regulation of G2/M phase progression (Brehm et al., 1999; Wu et al., 2003; Macaluso et al., 2005). Accordingly, many RB interactors have been identified, including chromatin-modifying proteins such as histone deacetylases (HDACs) (Luo et al., 1998) and histone methyltransferases (Nielsen et al., 2001) [for review, see (Dick and Rubin, 2013)]*.* Key interactions are mediated by the large pocket domain, encompassing residues 379–928 (Sellers and Kaelin, 1997). This domain harbors two conserved interaction interfaces, one that is typically engaged by E2F TFs and a binding cleft that has been shown to bind an LxCxE consensus sequence present in viral oncoproteins such as the SV40 large T-antigen, adenovirus E1A and human papilloma virus (HPV) E7 protein (Lee et al., 1998; Kim et al., 2001). Interestingly, the latter interaction site was shown to be important for RB interactions on chromatin, e.g., with HDACs (Brehm et al., 1998; Isaac et al., 2006). Beyond the conserved LxCxE RB interacting motif, surrounding residues and other interaction interfaces contribute to the association of individual proteins with RB (Singh et al., 2005).

The interaction of KDM5A with RB is mediated through two possibly independent sites: its LxCxE motif (LFCDE in KDM5A, aa 1373–1377) and a part loosely termed non-T/E1A region (NTE1A), located C-terminal of the LxCxE motif (Kim et al., 1994). The NTE1A nomenclature indicates that this binding site differs from the classical sites on RB targeted by viral proteins. The cellular interaction of RB and KDM5A remained difficult to demonstrate for some time, but was eventually confirmed by co-immunoprecipitation and detected within transcriptionally active regions during cellular differentiation (Benevolenskaya et al., 2005). KDM5B has a strong overall similarity with KDM5A, including an identical distribution of protein domains (Figure 1B). Accordingly, KDM5B also interacts with RB in cells, but lacks an LxCxE consensus sequence for RB binding. Instead, the NTE1A region of KDM5B is required for RB interactions in cells, which was suggested to stabilize hypophosphorylated RB (Roesch et al., 2005). In agreement with this observation, KDM5A colocalized with RB in regions enriched for hypophosphorylated RB (Benevolenskaya et al., 2005). Interactions of KDM5C or D with RB have, to our knowledge, so far not been observed. It is unclear in how far the functional relationship with RB is conserved throughout the KDM5 family.

The interplay of RB/KDM5 has particularly been studied in the contexts of cancer and differentiation. In melanoma, where slow-cycling cancer cells show high KDM5B expression, RB/KDM5B interactions may be involved in tumor suppression (Roesch et al., 2006). In both cancer and developmental contexts, phenotypes caused by RB dysfunction could be rescued by inhibiting KDM5A, leading to the hypothesis that at least part of the functional link between RB and KDM5A may be based on antagonizing roles (Benevolenskaya et al., 2005; Lin et al., 2011). For example, interfering with KDM5A expression or inhibiting its demethylase activity reduced tumor initiation and growth in RB-deficient mice, significantly expanding life span (Lin et al., 2011; McBrayer et al., 2018), and decreased cellular heterogeneity in a small cell lung cancer (SCLC) cell line (Varaljai et al., 2015). In RB-deficient SCLC, KDM5A activity was shown to be required for the maintenance of the neuroendocrine phenotype and to promote cancer cell proliferation (Oser et al., 2019). These observations could be explained by an inhibitory effect of RB on histone demethylation by KDM5A, either in a direct or indirect manner, and highlight the therapeutic promise of inhibiting KDM5 demethylases, e.g., in RB-deficient cancers.

It was suggested that RB functions that promote cellular differentiation and transcriptional activation are independent of its interactions with E2F TFs, instead requiring its association with KDM5A (Sellers et al., 1998; Benevolenskaya et al., 2005; Lin et al., 2011) with some evidence thus suggesting a role of KDM5A as a transcriptional activator (Benevolenskaya et al., 2005). More recent studies suggest that the release of the transcriptional repression of metabolic regulators by KDM5A may be responsible for the restoration of differentiation upon KDM5A knock-out in RB-deficient cells (Varaljai et al., 2015). Altogether, studies on the relationship of KDM5A and RB in RB-dependent differentiation pathways indicate that the immediate effects of RB/KDM5A complexes on transcriptional activity depend on the target genes, involving divergent mechanisms that may imply either antagonistic or synergistic effects between these regulators (Benevolenskaya et al., 2005; Lopez-Bigas et al., 2008; Varaljai et al., 2015).

In the context of cellular senescence, evidence suggests that RB functionally cooperates with KDM5A and KDM5B to promote cell cycle arrest and senescence phenotypes. Here, upon down-regulation of RB, an increase of H3K4me3 levels was observed at RB-dependent E2F target genes and the loss of H3K4me2/3 at E2F target genes during senescence induction was dependent on KDM5A demethylase activity and its RB binding region (Chicas et al., 2012). A similar functional relationship was determined in a mouse embryonic fibroblast model of cellular senescence (Nijwening et al., 2011), suggesting a common and potentially redundant (Chicas et al., 2012) role of KDM5A and KDM5B in RB-dependent senescence induction. Of note, these observations hinting at a localized correlation of RB binding and KDM5-dependent H3K4 demethylation would not be immediately incompatible with the idea that RB inhibits KDM5 demethylase activity, suggesting that the RB/KDM5 interplay may depend on the experimental model and biological pathway. Also, the latter findings focus on E2F-dependent RB targets, whereas other studies on the RB/KDM5A axis during cellular differentiation (Benevolenskaya et al., 2005; Lopez-Bigas et al., 2008; Varaljai et al., 2015) consider E2F-independent functions of RB. It should be noted that diverse mechanisms may affect the distribution of H3K4me3, including histone methyltransferases or nucleosome remodelers, complicating direct causal conclusions in complex cellular systems.

In summary, there is compelling evidence of direct interactions and a close interplay of RB and KDM5 demethylases, in particular KDM5A and B. Both a synergistic relationship, e.g. during senescence induction, and the mutual inhibition of catalytic activity and regulatory functions have been suggested. It seems that the biological context plays an important role in determining the manifestations of this cross-talk. Given the significance of RB and KDM5 demethylases in development and disease, mechanistic studies will be of great interest to elucidate the molecular basis of these associations and their regulation. It will be of key importance to decipher which implications are mediated by the function of stable RB/KDM5 complexes, and which are the consequences of altered RB and KDM5 functions and activities. For example, it is unclear whether KDM5/RB complexes can bind and demethylate nucleosomes, and how they are recruited to their genomic targets. Since the functions and mechanisms of KDM5/RB complexes seem to vary significantly, elucidating the molecular determinants of RB interactions with different KDM5 family members and in distinct contexts will be of particular importance. Moreover, since the demethylase activity of KDM5A and B underlies their tumor-promoting roles (Vinogradova et al., 2016) and KDM5A/B inhibition is particularly promising in RB-deficient tumor cells (Oser et al., 2019), a potential mechanism of KDM5 inhibition by RB may lead the way towards novel approaches to interfere with oncogenic activities of KDM5 demethylases in defined contexts. Interestingly, while phosphorylation is the best known PTM regulating RB function, other PTMs such as lysine methylation also contribute to RB regulation (Munro et al., 2010; Saddic et al., 2010; Carr et al., 2011; Cho et al., 2012). To our knowledge, non-histone substrates of KDM5 enzymes have so far not been discovered, leaving open the question whether RB demethylation is a possible mechanism underlying the RB/KDM5 interplay.

### Histone Deacetylase Complexes

Regulatory complexes interact physically and functionally on chromatin, coordinating their catalytic activities and recruitment. These interactions provide a complex framework for the local, context-dependent reshaping of chromatin (Blackledge et al., 2014). Understanding the interplay of KDM5 enzymes with epigenetic multi-protein complexes may provide valuable clues regarding their distinct cellular functions despite a similar domain organization (Christensen et al., 2007; Klose et al., 2007; Lee et al., 2007). Numerous studies report on such interactions, with histone deacetylase (HDAC)-containing complexes consistently shown to physically associate with KDM5 demethylases. Most HDACs, just like many chromatin modifying enzymes, reside within larger multi-protein complexes that regulate histone lysine acetylation levels (Seto and Yoshida 2014; Park et al., 2020). Histone acetylation facilitates chromatin dynamics or recruits regulators via reader domains such as bromodomains, ultimately promoting transcriptional activity (Zeng and Zhou, 2002). Consequently, histone deacetylation is associated with transcriptional repression (Hu et al., 2000; Huang et al., 2000), suggesting a functional overlap with H3K4 demethylation. Available evidence suggests that the dynamic association of KDM5 demethylases and HDAC complexes on chromatin contributes to their genomic targeting, thereby potentially coordinating H3K4 demethylation and histone deacetylation, leading to transcriptional repression (Hayakawa et al., 2007). KDM5 enzymes were shown to interact with three major HDAC complexes: the nucleosome remodeling and deacetylase (NuRD), SIN3B-containing, and CoREST complexes.

The NuRD and SIN3B-containing HDAC complexes are key chromatin regulators associated with transcriptional repression (Silverstein and Ekwall, 2005; McDonel et al., 2009). While they share the core components HDAC1/2 and RBBP4/7, they differ in their additional subunits, with SAP18/30, SDS30, MRG15 (MORF4L1), EMSY, GATAD1 and PHF12 as part of SIN3B-containing complexes (Grzenda et al., 2009; Varier et al., 2016) and CHD3/4, MBD2/3 and MTA1/2/3 present in NuRD complex variants (Seto and Yoshida, 2014; Millard et al., 2016). Using immunoprecipitation and density gradient fractionation, FLAG-tagged KDM5A was shown to associate with subunits of both the NuRD and SIN3B complexes. The detected assemblies could be physically separated and their co-precipitation with KDM5A was differentially disrupted by deletions of KDM5A, hinting at distinct interfaces selecting for interactions with either the SIN3B or the NuRD complex (Nishibuchi et al., 2014). A suggested interactor of both KDM5A and NuRD, Zinc finger MYND domain-containing protein 8 (ZMYND8), links the recruitment of KDM5A and the NuRD complex to sites of DNA damage, suggesting a role of KDM5A beyond transcriptional regulation (Gong et al., 2017). Interestingly, ZMYND8 was also reported to directly interact with KDM5C (Shen et al., 2016) and KDM5D (Li et al., 2016b), contributing to their genomic localization and functionally cooperating with these KDM5 enzymes. Both reports, however, suggest the ZMYND8-mediated recruitment of KDM5C and D to different genomic elements, namely enhancers (Shen et al., 2016) and transcription start sites (Li et al., 2016b), respectively. The molecular cues that specify these apparently divergent recruitment events have so far remained unclear. Also, it is not known in the case of KDM5C and KDM5D whether the association with ZMYND8 also implies interactions with HDACs or other chromatin regulators such as NuRD. The physical association of KDM5B with the NuRD complex subunits MBD3, LSD1 and HDAC1 was shown using immuno-purification approaches (Li et al., 2011). Additional studies verified the interaction with HDAC1 and further ChIP analysis revealed that KDM5B colocalizes with NuRD complex subunits on chromatin (Klein et al., 2014).

Immunoprecipitation experiments identified KDM5A to directly interact with MORF-related gene on chromosome 15 (MRG15/MORF4L1), a subunit of SIN3B complexes (Hayakawa et al., 2007). Large-scale proteomics studies strongly support KDM5A being a stable component of complexes that include SIN3B, MRG15, HDAC1/2, RBBP4/7, as well as PHF12, EMSY (c11orf30), and GATAD1 (Vermeulen et al., 2010; Malovannaya et al., 2011). The association with this complex facilitates KDM5A recruitment to specific genomic loci, in particular promoter regions with high levels of H3K4me3. Interestingly, genomic occupancy of this KDM5A-containing complex was associated with transcriptional activation of a subset of genes, with an enrichment of pro-proliferative genes. The involvement of KDM5A demethylase activity was not investigated in this study (Varier et al., 2016). ChIP-Seq analyses suggested that KDM5B and the *Drosophila* KDM5 homolog Lid also interact with MRG15, a chromatin organizer that binds methylated histone H3K36me3 (Zhang et al., 2006), leading to KDM5B and Lid recruitment to H3K36me3-bearing regions (Moshkin et al., 2009). Further studies on Lid support the notion that a functional interplay between KDM5 demethylases and SIN3 HDAC complexes may be evolutionarily conserved. In biochemical studies Lid was copurified with the HDAC1 homolog RPD3 as part of a larger multi-protein complex that also contained MRG15. This interaction did not affect the catalytic activity of Lid while having an inhibitory effect on RPD3 HDAC activity (Lee et al., 2009; Di Stefano et al., 2011). Functional and biochemical analyses further support the idea that SIN3 and Lid cooperate in transcriptional regulation during development (Gajan et al., 2016). Since KDM5C or D have not been detected so far as interactors of the above SIN3B complexes, this mechanism may be a distinguishing feature among KDM5 family members in mammals.

The CoREST complex is a transcriptional repressor of neuronal and stem cell fate genes, consisting of the RE1-silencing transcription factor (REST), HDAC1/2, lysine-specific demethylase 1 (LSD1/KDM1A) and RCOR1/2/3 (Wang et al., 2007; Foster et al., 2010; Song et al., 2020). CoREST was co-purified with affinity-tagged KDM5C (Tahiliani et al., 2007; Nishibuchi et al., 2014), and chromatin immunoprecipitation (ChIP) analyses with REST coupled with KDM5C depletion experiments showed overlapping genomic targets (Tahiliani et al., 2007). Biochemical analysis of KDM5C showed no significant changes in enzyme activity in this context. In agreement with these findings, dysregulation or mutations of either KDM5C or REST are linked to neuronal disorders such as X-linked intellectual disability, autosomal recessive intellectual disability and autism (Santos et al., 2006; Najmabadi et al., 2011).

LSD1 (KDM1A) stands out as another potential interactor of KDM5A since its lysine demethylase activity targets the same histone H3 residue as KDM5 demethylases. As opposed to KDM5 enzymes, however, demethylation by LSD1 is restricted to H3K4me1 (Shi et al., 2005). It is therefore a tempting idea that KDM5 demethylases and LSD1 may cooperate to fully demethylate H3K4. Indeed, ChIP analyses of KDM5B support the notion that both demethylases function cooperatively in the context of NuRD to demethylate H3K4 (Li et al., 2011). A large fraction of genomic regions in mouse ESCs occupied by KDM5B was found to be co-occupied by LSD1 and vice versa, supporting a partial and context-dependent co-operation of both enzymes (Kidder et al., 2014). However, direct experimental evidence of cooperative demethylation by KDM5 demethylases and LSD1 is lacking. LSD1 shares key interactors with KDM5 demethylases, e.g. by interacting with the CoREST and NuRD complexes in some contexts (Wang et al., 2009; Pilotto et al., 2015; Song et al., 2020). Cross-talk between these two enzymes may therefore take place within the molecular framework of larger multi-subunit complexes.

Genetic analyses of knock-out experiments suggested that interactions of LSD1 and the *Drosophila* KDM5 homolog Lid have variable implications depending on the chromatin environment. On one hand, Lid antagonized LSD1 silencing function and limited the spreading of heterochromatin beyond euchromatin-heterochromatin boundaries. On the other hand, both enzymes seemed to function cooperatively in the context of regulating Notch target genes by synergistically removing H3K4 methylation marks (Di Stefano et al., 2011). KDM5A was also shown to associate with the Recombination signal Binding Protein for immunoglobulin kappa J (RBP-J) co-repressor complex (Liefke et al., 2010), further supporting the link between KDM5A and Notch signaling, since the RPB-J corepressor complex is an important negative regulator of the Notch pathway, which controls important cell fate decisions. Interestingly, a functional interplay of RBP-J complexes with SIN3B- and MRG15-containing HDAC complexes is involved in the control of Notch signaling in *Drosophila melanogaster* (Moshkin et al., 2009; Liefke et al., 2010), supporting the links between SIN3B, HDACs and KDM5 demethylases. It will be interesting to see whether the involvement of KDM5 demethylases in a conserved pathway such as Notch signaling is also reflected on the molecular level in conserved interactions and molecular mechanisms. In support of a conserved role of KDM5 in Notch signaling, KDM5A repressed Notch dependent neuroendocrine differentiation in SCLC (Oser et al., 2019).

Another context in which KDM5 interactions with HDACs have been described is the transcriptional control of the circadian clock, where KDM5A was shown to be involved through direct interactions with the transcription factors Circadian locomotor output cycles protein kaput (CLOCK) and aryl hydrocarbon receptor nuclear translocator-like protein 1 (ARNTL, also known as BMAL1). Additional results suggested that KDM5A in complex with CLOCK and BMAL1 inhibits HDAC1 activity (DiTacchio et al., 2011). For HDAC4 and other class IIa HDACs, some experimental evidence suggests a possible interaction with KDM5B in the context of breast cancer and other cell lines (Barrett et al., 2007).

**FIGURE 4:**
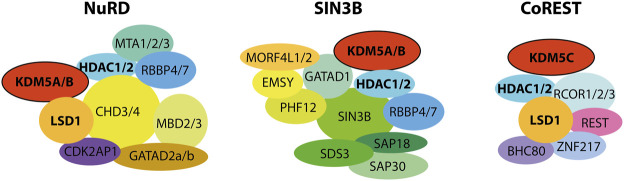
KDM5 demethylases were shown to physically and functionally interact with HDAC complexes. Interactions with the NuRD and SIN3B complexes have been shown for the mammalian KDM5A and B proteins, as well as *Drosophila* Lid. CoREST interactions were shown for KDM5C, with implications for neuronal development. Note that, for reasons of clarity, the stoichiometry and detailed subunit composition of the complexes was neglected. For NuRD and SIN3B, composition and dynamics of subunits are subject to research and have not been definitely established. The placement of subunits and their proximity to each other and to the KDM5 proteins does not reflect experimentally verified proximity within the respective complexes.

Taken together, a large body of evidence supports a physical and functional association of KDM5 demethylases with HDAC containing complexes, in particular NuRD, SIN3B and CoREST (Figure 4). It can be assumed that these interactions shown to impact KDM5 targeting and regulation are determinants of the diverse functions of individual KDM5 family members. For example, it is conceivable that KDM5C preferably interacts with CoREST, whereas KDM5A and B interact with NuRD and SIN3B (Figure 4). More detailed and targeted studies will have to be designed to address this hypothesis in the future. Other key open questions regard the interfaces within and between the respective complexes, defining which proteins and protein domains are directly involved. For example, it is not clear whether HDAC1/2, common catalytic subunits of KDM5 interacting complexes, are direct interactors stabilizing the association. Moreover, it will be pivotal to investigate the potential mutual regulation and coordination of demethylase and HDAC activities, as well as how interactions affect chromatin binding and genomic targeting. All of these questions require that detailed biochemical and structural studies are performed to pinpoint the molecular foundations of this regulatory interplay. Additionally, targeted functional studies will be required complementary to these mechanistic approaches to shed light on the implications within cellular and organismic contexts.

### Other Epigenetic Regulators

In addition to HDAC complexes, other epigenetic regulators likely contribute to KDM5 function through direct interactions. For example, a direct and functional interaction of KDM5A with Polycomb Repressive Complex 2 (PRC2) showcases the complexity of epigenetic regulation (Pasini et al., 2008). PRC2 is a key chromatin regulator that catalyzes the methylation of H3K27, resulting in the H3K27me3 mark associated with silent chromatin domains (Uckelmann and Davidovich, 2021). In particular, bivalent developmental genes, i.e. bearing both H3K4 and H3K27 methylation marks, are targets of both KDM5A and PRC2 binding. This interaction may suggest that PRC2 recruits KDM5A to target genes, but could also represent a basis of a coordinated demethylation of H3K4 and trimethylation of H3K27, ultimately promoting gene silencing (Pasini et al., 2008). In agreement with this, knock-out of KDM5B results in phenotypes reminiscent of Polycomb defects, pointing at a potential functional relationship (Albert et al., 2013). This functional cooperation, however, does not necessarily have to require physical interaction. The lysine methyltransferase KMT1C is generally considered to repress transcription by methylating H3K9me1/me2. *Via* co-immunoprecipitation KDM5A was identified as a binding partner of KMT1C. Similar to PRC2, KMT1C was suggested to stabilize KDM5A binding to chromatin and promote a coordination of enzymatic activity, resulting in transcriptional repression (Chaturvedi et al., 2012). KDM5D is the least studied KDM5 demethylase overall. Nonetheless, purification of FLAG-tagged KDM5D revealed a direct association with Polycomb group RING finger protein 6 (PCGF6), which is a component of non-canonical PRC1 complexes. Interestingly, it was shown that upon binding PDGF6, the demethylase activity of KDM5D was increased (Lee et al., 2007).

### Transcription Factors

In addition to interactions between epigenetic regulators mediating their context-dependent function, KDM5s are recruited to specific genomic sites by sequence-specific transcription factors (TFs). TFs can function individually or cooperatively, and can recruit further effector proteins (Spitz and Furlong, 2012; Lambert et al., 2018). Patterns of chromatin occupancy by KDM5 demethylases in various cell types indicate that TFs may directly recruit KDM5s to target genes (Varier et al., 2016). Accordingly, KDM5B was shown to bind the TFs PAX9 and BF-1 (also known as FOXG1b) in yeast two-hybrid interaction assays and LMO2 in Co-IP experiments. Generally, these proteins function as transcriptional repressors, playing a pivotal role in embryonic tissue and progenitor cell proliferation, respectively. KDM5B significantly increased the transcriptional repression in biochemical assays, corroborating the potential functional implications of these interactions (Tan et al., 2003; Roesch et al., 2008). While the mechanism was not explicitly stated, it is conceivable that PAX9, BF-1 and LMO2 may recruit KDM5B to genomic target sites, but also locally modulate its demethylase activity. Moreover, KDM5B is a co-regulator of various nuclear receptors, such as estrogen receptors, androgen receptors and progesterone receptors (Krishnakumar and Kraus, 2010; Catchpole et al., 2011; Vicent et al., 2013; Klein et al., 2014). KDM5C was shown to co-immunoprecipitate with the TFs c-Myc and ELK1, and c-Myc interactions were also detected for KDM5B and C upon their overexpression (Outchkourov et al., 2013). C-Myc had been described as a functional binding partner of KDM5A and KDM5B, as well as Lid (Secombe et al., 2007). In multiple myeloma, KDM5A was shown to support c-Myc-dependent transcriptional activation, although through an indirect mechanism mediated by direct interactions with the transcription machinery (Ohguchi et al., 2021). Clearly, TFs play an important role in specifying the localized activity and function of KDM5 demethylases. How TFs perform this recruitment function, and whether they exclusively bind to KDM5 proteins directly or within the context of larger, chromatin associated regulator complexes, remains to be studied in detail.

## Discussion

Histone demethylases of the KDM5 family display properties characteristic of many epigenetic regulators, making their exploration both challenging and fascinating. Functionally, KDM5 demethylases play diverse and seemingly contradictory roles that strongly depend on the biological context. For example, besides their repressive effect on transcription mediated by H3K4 demethylation, KDM5 demethylases can also facilitate transcriptional activation. Catalytic activity is directly responsible for some, but dispensable for other functions. Ongoing discussions regarding the direct causal effects of histone PTMs such as H3K4 methylation on transcriptional regulation (Cruz et al., 2018; Rada-Iglesias, 2018), and the requirement or dispensability of the activity of chromatin modifying enzymes (Dorighi et al., 2017) illustrate that fundamental processes in epigenetics still require clarification. Finally, dynamic interactions and, most probably, the regulatory interplay with chromatin features such as DNA, histone PTMs, as well as other chromatin associated regulators, define the contexts in which KDM5 demethylases perform their diverse roles. In order to decipher these roles, the molecular foundations of chromatin association and the molecular interactions and cross-talk of KDM5 enzymes with their interaction partners in the chromatin context have to be defined and mechanistically understood.

Over the last years, it has become clear that chromatin-associated processes are mediated by an intricate and dynamic interplay of proteins and their assemblies. Chromatin-modifying enzymes take part in these processes and have to be regulated such that their activity is locally and temporally defined. Establishing the underlying mechanisms is a key challenge towards elucidating the function of chromatin modifying enzymes. Mechanistically, this challenge comes down to deciphering the molecular cues that constitute a biochemical environment instructing catalytic regulation. Chromatin modifying enzymes are typically part of multi-subunit complexes harboring subunits that exert regulatory and targeting roles. Well-known examples are NuRD and PRC2 (Margueron and Reinberg, 2011; Allen et al., 2013). In these cases, subunit composition is one determinant of context-dependent activity, creating a dazzling complexity of regulatory mechanisms that are only beginning to be understood in molecular detail (Poepsel et al., 2018; Kasinath et al., 2021). In contrast to the above examples, KDM5 demethylases have not been described as constitutive members of multi-subunit complexes and it is not clear whether their cellular function strictly relies on their incorporation into such complexes. However, the experimental evidence reviewed here clearly shows that KDM5 function is intricately linked to other regulatory factors on chromatin. Also, it has become clear that KDM5 demethylases perform diverse roles that depend on the biological context. Intermolecular interactions on chromatin are likely to define these contexts and thus are essential for understanding KDM5 function at a molecular level. We will next outline critical gaps in our knowledge, key questions and how they might be approached at different levels in future studies.

### Defining Molecular Context

As we have seen, KDM5 demethylases engage in various processes, located at different sites within the genome, and these functions are reflected in diverse molecular interactions. KDM5A being a key regulator of Notch signaling in SCLC (Oser et al., 2019) is one example illustrating the opportunities associated with deciphering the underlying mechanisms. An important aspect of future efforts will be to further explore which direct interactions take place where in the genome, or within a given process. Most commonly, interactors of KDM5 demethylases have been identified via immunoprecipitation, often in the context of ectopically expressed, affinity-tagged KDM5 proteins or interaction partners. Using this approach, it can be challenging to derive direct physical interactions, since the association may be mediated by co-precipitated proteins and therefore be indirect. Furthermore, a pool of KDM5 is isolated from cultured cells and, thus, the identified interactions may reflect a convolution of various contexts. Future studies should therefore aim at defining the KDM5 interactome in specific contexts, identifying direct physical interactions. Such efforts may be guided by a combination of modern proteomic approaches such as proximity biotinylation or cross-linking mass spectrometry (CL/MS). Using proximity biotinylation, interaction partners are labelled depending on their spatial proximity through the spatially restrained activity of biotinylating enzymes or short-lived, reactive biotinyl moieties (Ummethum and Hamperl, 2020). A key aspect of these approaches is the ability to detect potential interactions in the context of live cells, preserving transient interactions that may be disrupted by extraction and wash procedures. CL/MS is a field of rapid technological development that enables the determination of direct interactions. In CL/MS, interactions are mapped to individual amino acids that are covalently linked by a chemical cross-linking reagent with a defined linker length (Sinz, 2018). CL/MS can now be applied to complex samples providing insights at the interactomic level (Yu and Huang, 2018), but can also yield detailed information on the topology of endogenous, multi-subunit complexes when coupled to affinity purification approaches (Schmidt and Urlaub, 2017; Mashtalir et al., 2018). Importantly, key interactions may rely on the chromatin environment, e.g. through contacts with DNA, nucleosomes, or chromatin bound TFs, and might therefore be disrupted during extraction procedures associated with classical immunoprecipitation protocols. Advanced protocols aiming at elucidating interactions in the context of intact, endogenous chromatin provide promising starting points to further explore KDM5 interactions in their native environment (Lambert et al., 2012). It is very important that such approaches are complemented with each other and with additional methods in order to confirm these results, e.g. in a reconstituted, biochemical system or through functional cellular assays. Furthermore, investigating distinct KDM5 functions of course also requires robust cellular or *in vivo* systems that enable appropriate read-outs of these functions, as well as consequences of perturbing defined interactions (see below).

### Interactions and Regulatory Mechanisms

Detailed mechanisms are typically derived from structural and biochemical approaches that define interaction interfaces at high resolution, including conformational rearrangements of protein domains and allosteric regulatory effects on enzymatic activities. While there are first studies reporting the regulation of KDM5A and B activity through chromatin contacts (Klein et al., 2014; Longbotham et al., 2019), no direct regulatory interactions between KDM5 demethylases and other chromatin regulators have been demonstrated yet. The coordinated functions of KDM5 demethylases, RB, and HDAC complexes suggest that the underlying interaction may very well imply the regulation of demethylase activity or a mutual regulatory cross-talk between different chromatin modifying enzymes. Such direct relationships should be explored in detail using biochemical reconstitution approaches, allowing for the high-resolution structural determination of interfaces and the systematic analysis of enzyme kinetics. On the basis of these mechanistic insights, targeted experiments can be designed that manipulate defined interactions rather than knock-downs or the deletions of large portions of the proteins that likely disrupt their function at large. Furthermore, chromatin binding by KDM5 demethylases has not yet been defined. The size and flexibility of chromatin-associated complexes were main factors hampering detailed structural analyses in the past. The development of structural methods such as single-particle cryogenic electron microscopy in recent years has made such challenging complexes more and more amenable to structure elucidation. Structure-function studies on chromatin modifying complexes such as PRC2 have since revealed molecular details of their chromatin association, recruitment, and activity regulation (Poepsel et al., 2018; Kasinath et al., 2021). Given the clear implications of KDM5 demethylases in cancer, there is a strong need of elucidating regulatory and recruitment mechanisms of individual KDM5 demethylases to provide potential starting points for developing therapeutic approaches targeting distinct KDM5 members and their functions, particularly in cancer. Mechanistic studies on the targeted activity of KDM5 demethylases in the context of chromatin will also reveal the basis of localized demethylation in distinct genomic regions, thus explaining, for example, the H3K4 demethylation at enhancers or promoters, leading to opposing effects on the transcriptional activity of target genes (Outchkourov et al., 2013).

### Functional Implications

Finally, experimental systems for the investigation of KDM5 function have to be developed or further improved to enable mechanistic insights. For example, ChIP-seq or related approaches such as CUT&Tag allow for the detailed analysis of KDM5 occupancy within the genome, as well as the co-occupancy with other chromatin regulators and the distribution of chromatin marks such as histone PTMs. It will be critical to design experimental approaches that enable the acute and rapid manipulation of KDM5 function for the interrogation of their activity, chromatin occupancy, and function within defined time-frames, reducing pleiotropic effects imposed by approaches that, for example, depend on the selection of single cell clones lacking a KDM5 protein or expressing a mutant protein. Functional read-outs should deliver information that reflects these time-frames while providing insights at sufficient detail and confidence. With respect to the roles of KDM5 demethylases in disease, it would be of great value to link discrete processes and regulatory mechanisms to molecular disease phenotypes. Therefore, appropriate experimental models that faithfully recapitulate key pathological features have to be used to determine the impact of defined molecular interactions and mechanisms on disease processes and provide a testing ground for KDM5-centered therapeutic approaches.

## Conclusion

KDM5 demethylases are key epigenetic regulators involved in cellular differentiation, proliferation and development. These implications along with accumulating evidence suggesting KDM5 demethylases as promising targets in cancer therapy, call for a detailed investigation of the mechanisms that define their diverse functions. Targeting and regulatory interactions provide the molecular context in which KDM5 demethylases play their roles. RB and HDAC complexes are central interactors that coordinate with KDM5 demethylases in diverse ways. Future efforts will elucidate the molecular details and mechanistic implications of these interactions. Since RB is also an interactor of HDACs and HDAC complexes (Brehm et al., 1998; Lai et al., 2001), it will be of interest to determine whether RB takes part in HDAC interactions together with KDM5 demethylases. Finally, distinct interactions with chromatin regulators may not only define diverse functions of individual KDM5 demethylases, but could also provide hints to how these enzymes have diversified functionally within the KDM5 protein family. Taken together, these questions will continue to inspire novel experimental studies that will enhance our understanding of KDM5 demethylase biology and epigenetic mechanisms in general.
